# In Search of Hospice Information: Consumer Information Available on Hospice Compare and Yelp

**DOI:** 10.1089/pmr.2020.0022

**Published:** 2020-04-30

**Authors:** Anna N. Rahman, Susan Enguidanos

**Affiliations:** Leonard Davis School of Gerontology, University of Southern California, Los Angeles, California, USA.

**Keywords:** consumer-directed care, hospice, quality improvement, quality measurement

## Abstract

***Background:*** The hospice industry has expanded in recent years with limited oversight and few consumer-facing resources to assist consumers in selecting hospice agencies to care for their family members.

***Objectives:*** To better understand the availability of consumer-facing hospice information and how hospices are evaluated by these websites, this study examined two websites with national reach—the Centers for Medicare and Medicaid Services' Hospice Compare (HC) website and Yelp.com. We described Yelp hospice ratings and caregiver-reported ratings on HC and compared conceptually related HC ratings to each other.

***Methods:*** We collected hospice ratings from Yelp and hospice- and caregiver-reported quality indicators (QIs) from HC for all California hospices. We conducted descriptive statistics for all variables and conducted chi-square to examine differences in proportions for categorical variables. We conducted Pearson's correlation coefficient (*r*) to test the strength of the association between the hospice-reported pain assessment QI and the caregiver-reported indicators on HC.

***Results:*** Among our sample of 1040 California hospices, HC reported QIs for 200 (19.2%) hospices for the caregiver-reported QIs ranging to 448 (43.1%) hospices for the hospice-reported QIs. Just 236 hospices (22.7%) had a Yelp review. Hospice ratings on both Yelp and HC were fairly high. For-profit hospices were less likely to show HC QIs or to be rated on Yelp. Caregiver-reported HC ratings for pain and symptom management were significantly lower than conceptually related HC hospice-reported QIs.

***Conclusions:*** More research is needed to understand the lack of hospice representation on HC and investigate the usefulness of hospice-reported HC measures.

## Background

The number of hospices in the United States is increasing, as is the number of people who have used them. According to a recent report to Congress, “4,488 hospices in 2017 provided care to Medicare beneficiaries, a 2.4%increase from 2016, continuing more than 10 years of growth”^(p. 317)^ in the number of Medicare-certified hospices.^1^ At the same time—and again for the 10th year in a row—the number of Medicare beneficiaries receiving hospice services climbed, to ∼1.49 million in 2017, up 4.6% from 2016.^1^ In 2017, about half of the Medicare beneficiaries who died received hospice care.^1^

Amid the growing number of hospice agencies and patients served come increasing concerns about the quality of hospice care.^2–4^ A recent analysis for a 5-year period found that >3200 consumer complaints were filed with state officials, who identified problems among 759 hospices.^5^ A report by the federal office of the inspector general (OIG) found that “vulnerabilities in the Medicare hospice program affect quality care and program integrity,” namely that patients were not receiving the care promised and often suffered from poor pain and symptom control.^2^ Follow-up OIG studies found that most hospices have had at least one care quality deficiency and some hospices have inflicted serious harm on patients.^3,4^

The growing concerns around hospice quality have prompted the expansion of consumer-facing information about hospices' quality of care. Two online data sources with national reach are (1) Hospice Compare (HC), the government-administered website for standardized hospice quality measures, and (2) Yelp.com, one of the nation's most popular websites for consumer-reported business reviews.^6^ Both rating systems aim to help consumers make informed choices. Data from both are examined in this study.

### Hospice Compare

The Affordable Care Act (ACA) of 2011 mandated that the Centers for Medicare and Medicaid Services (CMS) establish a hospice quality reporting program (HQRP) that, among other aims, would help hospice agencies improve their services and help consumers evaluate and compare these agencies. The HQRP is a “pay-for-reporting” program; that is, simply reporting timely and complete data—as opposed to achieving a set level of performance on quality measures—determines compliance with HQRP requirements.^7^ Hospices are penalized financially for failing to submit required data. The ACA also required CMS to publicly report online “quality measures that relate to the care provided by hospice programs across the country.”^7^ As a result, in 2017, CMS launched HC.

HC data come from two sources: (1) quality indicator (QI) data submitted electronically by hospices to CMS and (2) surveys of caregivers, who cared for a patient who died under hospice care (certified independent evaluators conduct the surveys).^8^ Consumers can search the HC site for hospices by name or location and compare selected hospices.

### Yelp.com

Although other websites report consumer reviews of hospices, we focused in this study on Yelp.com because it is one of and by some accounts the most popular, trusted, and experienced consumer review website in the nation.^9,10^

Yelp.com allows consumers to post reviews of businesses and to rate them on a 1- to 5-star scale (5 is best). Yelp reports both the individual ratings and an average rating of all reviews for each business. Research in hospitals and nursing homes has found that Yelp reviewers tend to focus on subjective experiences of health care, such as their personal assessments of staff attitudes, rather than clinical aspects of care (e.g., pain management).^11,12^

Consumer review sites such as Yelp are widely known for both their strengths and weaknesses. Some online consumer reviews, for instance, are faked.^10^ Others are manipulated (e.g., businesses may encourage only satisfied customers to post reviews).^13^ The reviewer sample is not random. Consumers say they are aware of such problems, but also report that they trust these sites, perhaps because they are confident they can spot biased reviews.^13–15^

Recent trends show that consumers increasingly are using online review websites to select health care providers. According to a 2015 Healthcare Consumer Trends survey, 77% of consumers begin their health care search online, and 45% read online reviews before booking an appointment.^16^ Between 2008 and 2016, the cumulative number of health care-related reviews on Yelp jumped from 160,000 to 7.26 million,^4^ and then to 14.72 million in 2018.^6^ In 2019, health-related reviews comprised 8% of all Yelp reviews.^6^

### Study rationale

To date, no studies have examined HC and Yelp hospice ratings. These analyses could be of interest to myriad stakeholders, including patients and their families as well as hospice providers, payers, and regulators. This study aims to describe hospice data available from both Yelp and HC in California, including overlapping and unique factors for each site. Understanding the contribution from each rating site can inform efforts to empower consumers and enhance hospice care quality.

## Methods

We collected data in July and August of 2018 from three sources to conduct descriptive and comparative analyses of HC- and Yelp-reported quality ratings for California hospices. This study was part of a larger study that examined palliative care services in California. We focused on California because it leads the nation in number of Medicare hospice patients served and Medicare hospice expenditures. This research was exempt from Institutional Review Board approval because it used publicly available data.

### Data sources

#### Hospice agency data

We used data from the California Office of Statewide Health Planning and Development (OSHPD) to identify all California hospices.^17^ Licensed hospices submit annual utilization reports to OSHPD, which compiles them into a complete dataset. We used the 2016 dataset, the most current dataset available at the time to compile the initial list of licensed hospices and each hospice's ownership status.

#### HC data

For each hospice on the OSHPD list, we collected ratings from HC. For each Medicare-certified hospice in the nation, HC reports seven hospice-reported QIs that indicate the extent to which the hospice assesses patients' care preferences and treats common symptoms.^8^ We describe all seven hospice-reported QIs (Table 1).

**Table 1.** Descriptive Top-Box Data for Hospice Compare-Reported Measures for Hospices in California

Variables	No. of hospices with measure (% of all CA hospices)	Mean (SD)	Median
Patients or caregivers who were asked about treatment preferences such as hospitalization and resuscitation at the beginning of hospice care^a^	448 (43.1)	99.0 (4.9)	100
Patients or caregivers who were asked about their beliefs and values at the beginning of hospice care^a^	448 (43.1)	96.2 (11.0)	100
Patients who were checked for pain at the beginning of hospice care^a^	443 (42.6)	95.8 (7.8)	98
Patients who got a timely and thorough pain assessment when pain was identified as a problem^a^	338 (32.5)	86.1 (14.0)	90
Patients who were checked for shortness of breath at the beginning of hospice care^a^	441 (42.4)	100.0 (43.3)	100
Patients who got timely treatment for shortness of breath^a^	374 (36.0)	97.2 (3.8)	99
Patients taking opioid medication who were offered care for constipation^a^	247 (23.8)	93.1 (12.9)	99
Communication with family^b^	202 (19.4)	77.2 (6.6)	77
Getting timely help^b^	202 (19.4)	71.9 (8.5)	73
Treating patient with respect^b^	202 (19.4)	86.9 (7.3)	88
Emotional and spiritual support^b^	201 (19.3)	87.3 (4.5)	88
Help with pain and symptoms^b^	201 (19.3)	71.8 (6.0)	72
Training family to care for patient^b^	201 (19.3)	71.40 (7.47)	72
Rating of this hospice^b^	201 (19.3)	76.6 (7.1)	77
Willing to recommend this hospice^b^	200 (19.2)	80.6 (7.9)	81

Hospice Compare does not report these measures for hospices with <50 applicable cases.

^a^ Hospice-reported variable.

^b^ Caregiver-reported variable.

CA, California.

Since February 2018, HC also has reported results from a caregiver survey conducted monthly, with family and friends contacted at least two months after a patient's death.^18^ HC reports eight QIs derived from this survey, which we describe in Table 1.^18^

For both hospice and caregiver-reported indicators, HC reports the percentile of respondents giving the “top-box” response.^18^ Top-box scores report the percentage of all individual ratings that are the highest one or two possible ratings. In the case of HC's caregiver hospice rating, the top-box score reports the percentage of all individual ratings that are 9 and 10. Top-box responses for the questions comprising the pain-and-symptoms QI are “always” (as opposed to “usually,” “sometimes,” and “never”) received the help needed for pain and other symptoms. Hospices are exempt from the survey for any of several reasons, including (1) they newly started operation, (2) they had <50 survey-eligible caregivers in the previous year, and (3) an extraordinary experience beyond the agency's control occurred recently. More details about the survey, including a more detailed itemization of the survey exemptions, can be found at (www.hospicecahpssurvey.org/en/).

#### Hospice Yelp data

We also collected Yelp ratings for each hospice, focusing on only “recommended” ratings. (Yelp uses a software algorithm and user reports to filter out ∼25% of submitted reviews that are likely fake or manipulated to a “not recommended” section at the bottom of the listing.^19^)

We searched Yelp for ratings for each hospice on our list. If found, the agency was included in our dataset if its address on Yelp matched the agency's address as it appeared on the OSHPD list. For each included hospice, we recorded the overall star rating and the total number of individual reviews. We also recorded the total number of individual ratings for each star rating so that we could calculate the top-box score for each hospice, using the percentage of 5's as the top-box rating.

#### Analysis

We conducted descriptive statistics and frequencies for all hospice variables from both HC and Yelp. We also conducted chi-square tests to examine differences in proportions for categorical variables. We conducted Pearson's correlation coefficient (*r*) to test the strength of the association between the hospice-reported pain assessment QI and caregiver-reported indicators on HC.

## Results

### Sample description

We identified 1057 hospices certified by Medicare and/or Medicaid in California's 2016 annual facility utilization report.^17^ We excluded 17 (1.5%) hospices that had since closed.

Of the final sample of 1040 hospices, for-profit agencies comprised 823 hospices (79.1%). Nonprofit agencies comprised 75 hospices (7.2%). Compared with national prevalence rates of 69% and 27%, respectively, for hospices, our sample includes a higher percentage of for-profit agencies and a lower percentage of nonprofit agencies.^1^ Ownership status for 131 agencies (12.6%) was unavailable. The remaining 11 hospices were categorized as government-owned or “other.”

#### Results for hospices with HC measures

As shown in Table 1, HC reported QIs for a minority of the 1040 hospices included in this study, ranging from 200 (19.2%) hospices for the caregiver-reported QIs to 448 (43.1%) hospices for the hospice-reported QIs. Table 1 presents descriptive data for these QIs. In terms of hospice ownership, analysis among hospices with ownership information available (*n* = 909) showed that nonprofit hospices were significantly more likely to have HC-reported measures than for-profit hospices (74.4% vs. 46.7%, respectively; χ^2^ = 24.0; *p* < 0.001). The same pattern was found among HC caregiver-reported measures, with significantly higher portions of nonprofit hospices showing caregiver measures than for-profit hospices showing these measures (66.3% vs. 17.6%, respectively; χ^2^ = 106.7; *p* < 0.001).

#### Results for hospices with Yelp reviews

Table 2 presents descriptive results for hospices with Yelp reviews. A total of 236 hospices (22.7% of the total sample) had 1 or more consumer reviews on Yelp.com, with a mean of 6.5 reviews (SD = 8.4). The mean *overall rating* (i.e., the mean of the average rating per agency) was 4.0 (Table 2). There were 1520 individual Yelp ratings for these hospices, most commonly a rating of 5 (*n* = 1018, or 67.0%), followed by 1 (*n* = 368, or 24.2%). Less frequently used were ratings of 2 and 4 (*n* = 57 or 3.8%, and *n* = 53 or 3.5%, respectively), and, finally, 3 (*n* = 24 or 1.6%). The mean for the total sample of *individual* ratings was 4.0 (SD = 1.2).

**Table 2.** Descriptive Data for California Hospices with Yelp Reviews (*N* = 236)

	Yelp reviews	Overall Yelp rating
Mean (SD)	6.5 (8.4)	4.0 (1.2)
Median	3.0	4.5
Mode	1.0	5.0
Range	1–49	1–5

Similar to HC measures, nonprofit hospices were significantly more likely to have Yelp reviews than for-profit hospices (67.4% vs. 45.0%, respectively; χ^2^ = 15.8; *p* < 0.001). There were no significant differences in Yelp *ratings* by ownership status (for-profit: *M* = 4.0 [SD = 1.2], nonprofit: *M* = 3.9 [SD = 1.2]; *p* = 0.5).

### Comparison within HC: Caregiver-reported measures versus hospice-reported measures

Among hospices with the selected HC measures (Table 1), we examined the extent to which responses correlated between the caregiver-reported measure “(the agency) helped with pain and symptom management” rating with the three conceptually related hospice-reported QIs (“timely and thorough pain assessment” “timely treatment for shortness of breath,” and “offered care for constipation”).

The caregiver-reported measure was significantly but weakly correlated with the hospice-reported “timely and thorough pain assessment” measure (*r* = 0.142; *p* = 0.049). The correlations between the caregiver-reported rating and each of the other two hospice-reported measures (“timely treatment for shortness of breath,” *r* = 0.037, *p* = 0.614, and “offered care for constipation,” *r* = 0.102, *p* = 0.167) were weak and not significant.

## Discussion

This study is one of the first to examine availability of consumer-facing hospice information. Overall, we found that less than half of California hospices have hospice-reported HC data available. This number was even lower for caregiver-reported HC measures, with <20% of hospices represented. Similarly, Yelp data were available for just 22.7% of California hospices.

### HC-related findings

The percentage of hospices reporting each of the HC-published QIs was lower than the percentages anticipated in initial QI testing, which estimated that 70% to 90% of all hospices would report QIs if the minimum number of stays required for reporting any one QI was 20.^20^ We also found that for-profit hospices were less likely to show HC QIs than not-for-profit hospices. This finding is in keeping with findings from a recent study by Hsu et al. that hospices electing not to submit HC data to CMS were more likely to be for-profit agencies. That study also found that nonparticipants also had lower nurse staffing ratios and nonaccreditation.^21^ Other studies have found that for-profit hospices are more likely to engage in questionable care practices.^21–23^ Considered together, these findings, from both our and other recent studies, raise concern that some for-profit hospices may be purposefully withholding HC data that reflect poorly on their care practices, despite the fact that they incur a financial penalty for failing to report HC data. Given evidence that for-profit hospices are replacing not-for-profit hospices in an expanding industry,^24^ we agree with Hsu et al. that there is “an urgent need to address (a possible) selection bias in quality reports.”^21^^(p. 33)^

Also worth noting is that all but one (“pain assessed”) of the hospice-reported QIs had mean ratings >93%, with median ratings ranging from 98% to 100% (again excluding the “pain-assessed” QI). In general, the usefulness of health care measures diminishes as their ratings approach perfect (i.e., 100% on HC), for such high ratings no longer reveal meaningful differences among the reporting health care agencies. CMS recently decided not to discontinue any of the QIs on the HC website^25^; we recommend, however, that it consider retiring what appears to be topped-out measures.

Even fewer hospices had caregiver-reported QIs on HC than hospice-reported QIs. This is regrettable in that health care consumers have reported that, when searching for a new provider, they want to read about the experience others have had with the provider.^26^

Although not a primary aim of this article, we explored the HC website in depth for this research and spent considerable time following numerous links to read technical documents to understand how HC data are collected and scored.^18^ This was far from the “user friendly” experience CMS intended.^27^

### Yelp-related findings

Similar to caregiver-reported QIs, just under a quarter of California hospices had one or more Yelp reviews. Hospices and patient and consumer advocates should encourage hospice users to post social media reviews of their providers, for health care consumers consult these websites, tend to trust social media reviews (and their own ability to spot fake or manipulated reviews), and, when surveyed, say they want to see more of this type of feedback.^28^

The overall average Yelp rating for hospices was a relatively high 4.0; however, other studies have found that a business's initial online consumer ratings can be volatile, and possibly upwardly biased.^29^ The number of reviews at which stabilization occurs has not been determined. Experts have speculated, however, that the number is considerably higher than the average (*N* = 6.3) found in this study.^29^ In addition, the distribution of individual hospice ratings on Yelp was bimodal and polarized, with mostly 5's (69.9%) and 1's (23.2%). These results challenge the common assumption among business providers that disgruntled consumers post most Yelp reviews, meting out low ratings.^30,31^ In fact, Yelp reports 49% of all individual reviews are 5's and 19% are 4's (just 16% are 1's).^6^

### Hospice-reported measures versus caregiver-reported measures

The caregiver-reported QI about pain and symptom management did not correlate strongly with the three hospice-reported QIs that conceptually relate to this QI. In addition, the mean rating for the caregiver-reported QI was significantly lower than each of the three conceptually related hospice-reported QIs. This finding may be due to methodological differences in how the measures are constructed. The caregiver-reported measure for pain and symptom management, for instance, is a composite rating derived from top-box responses to four survey questions. Each of the three hospice-reported measures is derived from a single item.^8^ More research is needed to determine whether these discrepancies persist in larger samples, and, if so, the explanations for them.

Finding from previous studies suggest two additional possible explanations for our findings of higher ratings among hospice-reported QIs. First, previous studies have found that simply initiating quality measures improves outcomes.^26^ Thus, it may be that implementation of QI measures in hospice led to improved compliance with assessment of symptoms and treatment. Second, prior studies have found evidence that some nursing homes reported inflated measure data to CMS to increase their ratings on Nursing Home Compare.^32,33^ It may be that some hospices engaged in a similar practice and reported inflated measure data to CMS to improve their HC ratings.

Our findings may inform current efforts by CMS to add new measures to HC.^27^ One option may be to adopt select Palliative Care Outcome Scale measures developed in the United Kingdom and used internationally. These measures focus on identifying areas of concern from the patient perspective, including how much patients are affected by pain and symptoms. Standardized repetition of these metrics can serve several purposes:

1. Improve symptom identification
2. Better identify patient-centered goals
3. Facilitate communication
4. Improve treatment in response to identified problems
5. Provide QI data regarding the ability of the hospice to meet patient-centered outcomes.^26–28^

### Limitations

We collected data for only California hospices and our results may not be generalizable to other states. A recent study found that California has a lower HC participation rate than most other states.^21^ It also serves more Medicare hospice patients than other states.

## Conclusions

Hospice ratings on both Yelp and HC are available for a limited number of hospices, thus consumers visiting either site will find many hospice agencies lack the evaluation data they seek. Our study revealed encouraging findings (e.g., on the whole, hospice ratings on both Yelp and HC were fairly high) and concerning trends (e.g., an unusually high number of hospices missing HC measures and Yelp reviews, and a topping-out of hospice-reported HC measures). Future research is needed to understand the lack of hospice representation on HC and investigate the usefulness of hospice-reported measures. Finally, CMS may want to consider adopting more patient-centered outcomes in their QIs. In the meantime, consumers should be advised to consult both HC and consumer rating sites to inform their decisions about hospice selection.

## Abbreviations Used

CA: California
CMS: Centers for Medicare and Medicaid Services
HC: Hospice Compare
HQRP: hospice quality reporting program
OSHPD: Office of Statewide Health Planning and Development
OIG: office of the inspector general
QIs: quality indicators
